# Physical compared to mental diseases as reasons for committing suicide: a retrospective study

**DOI:** 10.1186/s12904-016-0088-5

**Published:** 2016-02-09

**Authors:** Martin Fegg, Sybille Kraus, Matthias Graw, Claudia Bausewein

**Affiliations:** Department of Palliative Medicine, University of Munich, Munich, Germany; Department of Forensic Medicine, University of Munich, Munich, Germany

**Keywords:** Physical disease, Mental disease, Suicide, Palliative care, Cancer, Suicide attempt

## Abstract

**Background:**

Several studies investigated the relationship between mental disorders and suicidal ideation. However, little is known about physical illnesses being the major trigger for committed suicides. It is necessary to understand these risk factors to be able to meet the needs of patients in a palliative care setting.

**Methods:**

Suicide, medical and police notes were retrospectively analysed from all autopsies conducted in 2009–11 at the University of Munich, Germany. Documented reasons for suicide were classified into a “physical disease” (PD) or “mental disease” (MD) group and compared with respect to their sociodemographic characteristics and autopsy outcomes.

**Results:**

Of all 1069 cases, 18.9 % gave a PD as reason for committing suicide (MD, 32.7 %). Those indicating PD were older than MD (68.8 vs. 48.7 years; *p* < 0.001) with more men being in this group (72.8 % vs. 59.1 %; *p*=0.002). In PD, 30.7 % suffered from cancer, 28.7 % from chronic pain and 12.4 % from lung disease. 38.8 % of MD and 12.4 % of PD had previous suicide attempts.

**Conclusions:**

In palliative care, it is necessary to screen patients on a regular basis for suicidal ideation, especially those with previous suicide attempts.

## Background

In 2012, about 869,600 people died in Germany with around 10,000 people committing suicide [1]. Thus, more people died from suicide compared to death as consequence of an accident. The risk for committing suicide is a complex phenomenon, including biological, psychological and socio-cultural factors: older age, male sex, previous suicide attempts, psychiatric illnesses (e.g. mood disorders, substance abuse, schizophrenia, personality disorders [2]) and negative life events (e.g. lovesickness, loss of a person/job, feeling of being isolated or not being accepted, financial problems) have been identified as underlying reasons [3–5].

Psychiatric disorders are estimated to be responsible for a large proportion of suicides (up to 70–100 %) [6, 7]. In palliative care and cancer patients, depression and hopelessness have been identified to be major triggers for the wish to hasten death as well as suicidal ideas [8–13]. Mood disorders explained the increase of suicide risk in patients suffering from coronary heart disease, stroke, and chronic obstructive pulmonary disease [14].

Functional disability, as well as a number of specific physical illnesses (e.g. lung cancer, gastrointestinal cancer, breast cancer, genital cancer, bladder cancer, lymph node cancer, neurological disorders, pain, chronic obstructive pulmonary disease, liver disease, male genital disorders, and arthritis/arthrosis), were shown to be associated with suicidal ideation in older adults [15, 16]. Cancer patients have a higher risk to develop major depression and their risk to commit suicide is doubled compared to the healthy population, especially during initial diagnosis and progressive disease. Margari et al. found that patients with chronic pain syndromes are more aggressive and impulsive in their behaviour [17]. These characteristics have been judged as important factors for (pre-)suicidal behaviour [18, 19]. Mosich et al. assume that about 10 % of all patients under palliative care suffer from suicidal ideation [20]. Filiberti et al. examined the characteristics of terminal cancer patients who committed suicide at home: patients’ burden was related to the fear of loss of autonomy, increasing physical dependence, worry to be a burden to the family and hopelessness in awareness of the limited prognosis [10].

However, physical illness is rarely investigated as a motive for committed suicides with the effects being unclear: e.g. cancer was associated with increased risk of suicide even after adjustment for all mental disorders [21].

The aim of this study was to evaluate i) how many people commit suicide with a physical illness as main underlying cause, ii) how this group differs from people committing suicide with a mental disorder given as reason and iii) which factors may be associated with suicide in physically ill in order to better identify patients in a palliative care setting.

## Methods

This is a retrospective study of suicide notes, medical and police charts of persons who committed suicide and were examined at the Department of Forensic Medicine of Ludwig-Maximilians-University Munich between 2009 and 2011. The fatalities came from the catchment area of the department (similar to the catchment area of the Higher Regional Court Munich), with the majority of suicides committed in the city and county of Munich (about 61 %).

### Data collection

In total, 1069 suicides were referred with the following information being gathered at the coroner’s office: 1) forensic results including autopsy, histological and toxicological chemical data, 2) criminal investigations and prosecutor’s investigative results. The files of the prosecutor's office were anonymously analysed with recorded permission and consisted of police investigations (e.g. death circumstances), hearings of witnesses (e.g. relatives, other close persons, treating physicians), suicidal notes and medical records. In the majority of cases, extensive information about the suicide motive was included. An encryption key was developed to summarise this complex information.

All cases were screened for physical (PD) or mental diseases (MD) being mentioned as core reason for the suicide. In cases with both a PD or MD being documented, the notes were extensively revisited in an iterative process by three of the authors (MF, SK, CB) and a final decision was made upon agreement between the authors.

Descriptive statistical analyses were used for frequencies, means and standard deviations. Differences between groups were calculated using Student’s *t*-test, Mann-Whitney’s *U*-test and chi^2^-tests. Odds Ratios (OR) as well as univariate analyses were calculated controlled for age, gender, living situation (alone/with others), marital status (single/with partner), way of suicide (soft/hard; a “soft” method is considered to be “less aggressive”, e.g. intoxication, breathing back, whereas a “hard” method is considered to be more aggressive, e.g. hanging, guns). The significant level was set at 5 %. In multiple tests, Bonferroni corrections were applied. IBM SPSS Statistics Version 22 was used for statistical analyses.

This study was approved by the Local Research Ethics Committee of Munich University (LREC no. 11–14).

## Results

In 1069 suicide cases, 202 persons (18.9 %) clearly indicated a PD as main reason for the suicide, and 350 persons (32.7 %) a MD. In 517 cases (48.4 %), no or other reasons were given (e.g. lovesickness, family quarrel, financial problems).

### Sociodemographic characteristics

The mean age in PD was significantly higher compared to MD. In PD, also significantly more men committed suicide, while in PD significantly more subjects were single or living alone (Table 1). In MD, the most prevalent disorders were depression (70.6 %) and schizophrenia (16.3 %) followed by anxiety (8.6 %), delusional disorder (5.7 %), bipolar (4.0 %), borderline (2.9 %), and eating disorders (1.7 %).

**Table 1:** Sociodemographic Characteristics

	Physical disease as reason for suicide	Mental disorder as reason for suicide	Difference
	*n* = 202	*n* = 350	
Mean age (SD)	68.8 years (SD 14.9)	48.7 years (SD 16.1)	t = 14.5, *p* < .001
range	12–105 years	16–92 years	
Gender (%)	Female: 56 (27.2 %)	Female: 143 (40.9 %)	*χ*2 = 9.6; *p* = 0.002
Living situation			
Living alone	68 (33.7 %)	143 (40.9 %)	*χ*2 = 3.8, *p* = 0.05
Living with somebody else	123 (60.9 %)	179 (51.1 %)	
Marital status			
Single	106 (52.5 %)	239 (68.3 %)	*χ*2 = 13.9, *p* < 0.001
In partnership	95 (47.0 %)	109 (31.1 %)	

### Physical disease as main reason for suicide

In PD, the most common diseases were cancer, chronic pain, and heart diseases with vasuclar disease, metabolic disorders, neurological and musculosceletal diseases also being reported (Table 2). Interestingly, in MD physical diseases were also identified from medical records and autopsy results. However, these were not mentionned as reason for the suicide. Therefore, the rate of (comorbid) physical diseases might be higher.

**Table 2:** Physical diseases in complete suicides (multiple categories possible)

	Physical disease as reason for suicide	Mental disorder as reason for suicide	Difference
	*n* = 202	*n* = 350	
**Heart disease**	**56 (27.7 %)**	**14 (4 %)**	***χ*** ^**2**^ **= 9.6,** ***p*** **= 0.002**
Lung disease	25 (12.4 %)	14 (13.3 %)	*χ* ^2^ = 0.004, *p* = 0.95
Vascular disease	55 (27.2 %)	25 (23.8 %)	*χ* ^2^ = 0.9, *p* = 0.36
**Cancer**	**62 (32.5 %)**	**8 (7.6 %)**	***χ*** ^**2**^ **= 23.2,** ***p*** **< 0.001**
Metabolic disorder	48 (25.1 %)	33 (31.4 %)	*χ* ^2^ = 1.4, *p* = 0.25
Musculosceletal system	45 (23.6 %)	24 (22.9 %)	*χ* ^2^ = 0.02, *p* = 0.89
Neurological disease	39 (20.4 %)	21 (20.0 %)	*χ* ^2^ = 0.007, *p* = 0.93
Chronic pain	58 (30.4 %)	20 (19.0 %)	*χ* ^2^ = 4.5, *p* = 0.03
Duration of disease			
< 6 months	22 (20.0 %)	8 (17.8 %)	
6 months to 2 years	21 (19.1 %)	5 (11.1 %)	
> 2 years	67 (60.9 %)	32 (71.1 %)	U = 2251.5, *p* = 0.30
Number of diseases			
• 1 disease • ≥ 2 diseases	**68 (33.7 %)**	**57 (16.3 %)**	**U = 8154.0,** ***p*** **= 0.002**
	**123 (60.9 %)**	**48 (13.7 %)**	

Comments: Heart disease = hypertrophic heart, heart failure, diseases of the coronary arteries etc. Vascular disease = hypertension, arterial obstructive disease, thrombosis etc

Metabolic disorders *=* endocrinologic disease (e.g. diabetes mellitus, hypothyreosis)

Bold: Significant after Bonferroni correction (different diseases: *p* < 0.00625)

Vice versa, in PD mental disorders were found in medical history although not given as a reason for suicide: depression (25.7 %), schizophrenia (1.0 %), anxiety (0.5 %), eating disorder (0.5 %), and delusional disorder (0.5 %).

There were no significant differences between the groups with regard to the duration of the disease, although significantly more persons in PD had two or more diseases.

### Univariate analyses

A univariate logistic regression showed significant differences between the two groups with regard to age (*p* < .001), gender (*p* = .001) and type of suicide (hard/soft) (*p* = .03). Marital status (single/partner, *p* = .90) and living situation (alone/with somebody, *p* = .18) did not reach significant levels. In PD, men were more likely to commit suicide (OR = 2.04) as well as older people (over 50 years, OR = 7.81). PD were more likely to choose a “soft method” for suicide (OR = 1.64), although “hard methods” were also most prevalent in this group.

### Circumstances of suicide

Regarding the type of suicide, MD chose significantly more often jumping in front of a car whereas persons in PD most often shoot themselves. MD had significantly more often previous suicidal attempts (Table 3).

**Table 3:** Circumstances of suicide

	Physical disease as reason for suicide	Mental disorder as reason for suicide	Difference
	*n* = 202	*n* = 350	
Soft suicide method	67 (33.3 %)	107 (30.7 %)	*χ*2 = 0.422, *p* = 0.516
Hard suicide method	134 (66.7 %)	242 (69.3 %)	
Suicide method			
Strangulation	35 (17.3 %)	88 (25.1 %)	*χ*2 = 4.518, *p* = 0.03
Intoxication	59 (29.2 %)	90 (25.7 %)	*χ*2 = 0.793, *p* = 0.37
**Shooting**	**41 (20.3 %)**	**9 (2.6 %)**	***χ*** **2 = 48.852,** ***p*** **< 0.001**
Breathing back	12 (5.9 %)	10 (2.9 %)	*χ*2 = 3.182, *p* = 0.07
Jump from height	34 (16.8 %)	80 (22.9 %)	*χ*2 = 2.838, *p* = 0.09
Sharp forces	15 (7.4 %)	21 (6.0 %)	*χ*2 = 0.427, *p* = 0.51
**Jumping in front of vehicle**	**4 (2.0 %)**	**35 (10.0 %)**	***χ*** **2 = 12.546,** ***p*** **< 0.001**
Vehicle against barrier	2 (1.0 %)	2 (0.6 %)	*χ*2 = 0.312, *p* = 0.58
Gas inhalation	3 (1.5 %)	12 (3.4 %)	*χ*2 = 1.830, *p* = 0.18
Drowning	16 (7.9 %)	18 (5.1 %)	*χ*2 = 1.710, *p* = 0.19
Electricity	1 (0.5 %)	3 (0.9 %)	*χ*2 = 0.233, *p* = 0.63
Thermic force	1 (0.5 %)	8 (2.3 %)	*χ*2 = 2.561, *p* = 0.11
**Previous suicide attempts**	**25 (12.4 %)**	**136 (38.8 %)**	***χ 2 = 51.369,*** ***p*** **< 0.001**

Bold: Significant after Bonferroni correction (suicide method: *p* < 0.0042)

## Discussion

Mental disorders as well as physical illnesses may lead persons to commit suicide. In all cases explored, every fifth person indicated PD as the main reason for committing suicide and every third person MD. One third of the PD group suffered from cancer and almost the same proportion of heart disease or chronic pain. Health care professionals, especially in palliative care, will therefore be regularly confronted with patients who have suicidal thoughts and finally might commit suicide, even without obvious depression or other mental diseases being diagnosed. Suffering from a life limiting disease has a huge impact on quality of life as a consequence of pain, increasing dependence and continuous need of drugs with some having severe side effects. Longer duration of the disease (80 % suffered from their disease at least several months if not years) might also play a role for committing suicide because of perhaps little perspective regarding the course of the disease, decreasing social contacts or fewer tasks in life [22]. However, adequate symptom management, relieving burden and improving or restoring quality of life, may improve many of these aspects.

Several studies have shown an association between suicidal ideas and mental disorders [2, 23–25]. Only few studies focus the higher suicidal risk of people with physical diseases [26–30]. Singhal et al. reported that people with chronic physical diseases have a higher suicidal risk mainly because of the higher prevalence of (reactive) depression [31]. In a study of Breitbart et al., 16 % of terminal cancer patients suffered from clinical depression and 17 % of terminal cancer patients had a concrete wish to die [9]. Depressive patients had a fourfold wish to die compared to those without depression. In our study nearly a quarter of PD suffered from a comorbid depression. Although not explicitly mentioned as cause for the suicide, this might have influenced suicide wishes: thus, assessment and management of depression and hopelessness are paramount to minimise suicidal ideation. However, if comorbid mental disorders in PD are excluded, 13.6 % (*n* = 145) of cases remain with no mental disorder being mentioned or diagnosed. Therefore, it is necessary to regularly screen patients for suicidal ideas. Risk assessment tools and scales cannot substitute the clinical interview [32]. Especially patients with previous suicide attempts (12.4 % of patients with PD in our sample) or other risk factors (older age, male sex, longer duration of the disease etc.) have to be identified to find out the underlying reasons for their wish to die and help them with best palliative care and psychological support.

The age difference between the PD and MD group in our study is probably related to the younger age when mental illnesses are diagnosed compared to physical diseases. In our study, female sex was higher in MD, but overall there was a male dominance in both groups being consistent with several other studies [17, 30, 33].

Significant differences between both groups emerged regarding living situation and marital status with more people living in a relationship in PD compared to MD. Partnership problems are often associated with mental disorders [34].

Notable, in MD certain physical illnesses such as metabolic disorders, muscosceletal and neurological diseases (e.g. epilepsy) were also prevalent. This could imply that suicidal ideation in MD was also influenced by physical suffering. In both groups, the proportion of suicides increased with the duration of the illness indicating that long lasting and increasing burden was followed by decreasing coping strategies.

In both groups about two thirds used “hard methods” to commit suicide (more aggressive, all methods but intoxication and inhalation). However, intoxication was the most common suicide method in both groups. This percentage is higher than previously reported by German statistics (19 % intoxications) [1]. The accumulation of intoxications might be related to patients with advanced physical or mental diseases having more access to drugs with potentially lethal outcome. Therefore, it is necessary to assure patient compliance to regular drug intake and maybe prescribe smaller packages.

In MD, significantly more previous suicide attempts (threefold) were described. It is known that prior suicide attempts have a higher risk for a successful suicide [25, 35]. MD could have a more acute burden of suffering whereas the more chronic course in PD could lead to later decisions for suicide. Following this hypothesis, it might be assumed that palliative care professionals will have more contact with patients with a higher suicidal risk because mainly patients with more complex situations are being treated in palliative care.

### Limitations

Reasons and triggers for committing suicide comprise a broad range of bio-psycho-social as well as spiritual and environmental factors. Therefore, it is difficult to reduce this complex phenomenon to a single major cause. Exploring, discussing and reflecting considerable amount of information accessible after suicide, the authors were able to identify in around half of cases a more or less “physical” or “mental” reason. However, this process might be biased by 1) incorrect assignment by the authors, 2) undocumented, not communicated reasons or unavailable information, 3) lack of introspection in suicidal persons which might lead to a “physical” reason given although there are also mental problems.

Retrospective studies are not able to investigate causal relations but have an exploratory intention. Record reviews might be biased by incomplete information (e.g. if diseases were necessarily terminal). However, we tried to get a picture as full as possible in gathering all relevant information from the forensic department, the police, the treating physicians, and relatives. Subsequent prospective studies with control groups investigating suicidal thoughts in the context of a physical disease (with or without mental or adjustment disorders) are needed to further examine the findings. However, studies on committed suicides will often need to use (retrospective) post-mortem designs [15].

## Conclusion

Patients with long duration of mental and physical disease have a higher risk to commit suicide. Whilst the risk for suicide is known in mental disease, professionals should be aware that patients with severe and chronic physical disease also carry a risk for committing suicide.

Regular screening, adequate symptom control, psychotherapeutic and pharmacological treatment of mental disorders like depression [36], prescription of smaller packages of potentially lethal drugs, and regular checks of drug use could be potential steps to prevent suicides [5].

## Abbreviations

MD: mental disorder
OR: odds ratio
PD: physical disease
SD: standard deviation
